# Editorial: CD8+ T-cells in HIV/SIV infection, prophylaxis, and therapy

**DOI:** 10.3389/fimmu.2023.1159452

**Published:** 2023-02-21

**Authors:** Barbara L. Shacklett, Marcus Buggert, Joana Dias

**Affiliations:** ^1^ Department of Medical Microbiology and Immunology, School of Medicine, University of California, Davis, Davis, CA, United States; ^2^ Department of Medicine, Division of Infectious Diseases, School of Medicine, University of California, Davis, Davis, CA, United States; ^3^ Center for Infectious Medicine (CIM), Department of Medicine Huddinge, Karolinska Institutet, Huddinge, Sweden; ^4^ Immunology Laboratory, Vaccine Research Center, National Institute of Allergy and Infectious Diseases, National Institutes of Health, Bethesda, MD, United States

**Keywords:** HIV, SIV, CD8+ T cells, antiretroviral therapy (ART), HIV reservoir, tissue resident memory CD8+ T cells, CAR T cells

Although HIV-1 is now a treatable chronic infection, an effective vaccine remains elusive, and while cure has been achieved in rare cases, no effective, safe, and scalable curative protocol has yet become available for widespread use. A “functional cure” would reduce the amount of virus in the body below the threshold of detection, allowing the recipient to stop antiretroviral therapy (ART) without viral rebound. While this may be more readily attainable than a “sterilizing” cure, which would completely eradicate the virus, neither is straightforward. The HIV-1 vaccine field has focused largely on eliciting broadly neutralizing antibodies, but evidence from well-studied cohorts of HIV-1 controllers - persons who experience viral suppression without ART - supports a role for CD8+ T-cells in long-term immune control of HIV-1. Similarly, proposed strategies for functional HIV-1 cure frequently emphasize targeting CD8+ T-cells to tissue reservoirs of viral replication. A collection of five research articles published in Frontiers in Immunology between May and December of 2022 focuses on the role of CD8+ T-cells in HIV/SIV infection, prophylaxis, and therapy.

Despite long-term, effective ART and robust adaptive immunity, HIV-1 persists in lymphoid tissue reservoirs, thus far preventing most attempts at viral eradication. Fardoos et al. examined tissue resident memory (T_RM_)-like CD8+ T-cells in tonsillar tissue of persons living with HIV-1 (PLWH) from South Africa. Tonsillar CD8+ T_RM_-like cells were located outside follicular germinal centers, and had higher levels of αE integrin (CD103) and CD69 as well as a distinct transcriptional profile when compared to their counterparts in blood. HIV-1-specific CD8+ T_RM_-like cells showed increased expression of canonical T_RM_ markers and PD-1 as compared to CMV-specific CD8+ T-cells in tonsil. CD8+ T_RM_ cells in lymphoid tissues may represent an attractive potential target for immunotherapeutic interventions designed to stimulate the host adaptive response and eliminate tissue viral reservoirs.

What properties of CD8+ T-cells are associated with immune control of HIV-1 in persons on antiretroviral therapy? In a study of 60 such individuals on ART for more than 2 years with undetectable blood HIV-1 RNA for at least 6-12 months, and a CD4 cell count > 250/μl, Hu et al. identified a subset of CD8+ terminally differentiated effector memory cells (T_EMRA_) in blood expressing CCL5/RANTES but not CCL4/MIP-1β. The cells were described as “virtual memory” or T_VM_, analogous to a similar subset previously defined in mice as CD44^hi^CD122^hi^CD49d^lo^. These cells inhibited HIV-1 *in vitro* in a CCL5-dependent manner and displayed robust cytotoxicity. Their abundance was inversely associated with levels of HIV-1 DNA and unspliced RNA, suggesting that this subset might be mobilized to develop strategies for functional HIV-1 cure.

The impact of long-term ART on the body’s T-cell repertoire has been challenging to study, as this requires longitudinal samples obtained before and during ART. Towlerton et al. evaluated the composition and diversity of the T-cell receptor β-chain (*TRB*) repertoire in blood samples from 30 PLWH collected over a mean of 6 years, before and after ART initiation. Their study utilized archival specimens, many of which were collected prior to the WHO recommendation for immediate ART upon HIV-1 diagnosis. They found that despite a significant improvement in diversity following ART initiation, *TRB* repertoires remained significantly more clonal and less diverse in PLWH, even after long-term ART, than in a control cohort of bone marrow donors. However, results also suggested a higher degree of repertoire diversity in post-ART compared to pre-ART repertoires. It will be important to compare these findings to longitudinal studies of PLWH who began ART immediately upon diagnosis.

A means of targeting and eradicating HIV-1-infected cells in tissue reservoirs may be afforded by chimeric antigen receptor (CAR)-T-cells capable of recognizing viral envelope proteins in the surface of HIV-1-infected cells. Building on previous work infusing CD4-MBL CAR/CXCR5-T-cells into SIV-infected rhesus macaques, Davey et al. found that unfortunately, the CAR-expressing T-cells did not persist *in vivo* beyond 28 days. Anti-CAR Immunoglobulin G (IgG) antibodies were produced in all animals treated with the T-cells. The anti-drug antibodies (ADA) were directed primarily towards the CD4 D1/D2 domains, and partially to the CD28 transmembrane region. The majority could induce an antibody-dependent, cell-mediated cytotoxicity (ADCC) response. This study demonstrates that an ADA response can occur even when using self-proteins, likely due to the creation of novel antigenic sites.

Finally, Fernandez et al. described a new viral inhibition assay (VIA) designed to quantify CD8+ T-cell mediated inhibition of a panel of 35 HIV-1 infectious molecular clones expressing a *Renilla reniformis* luciferase reporter gene. The clones were derived from a broad range of HIV-1 clades, risk groups, and geographic areas represented in IAVI’s Protocol C, a large cohort spanning 5 sub-Saharan African nations. The assay reproducibly demonstrated circulating CD8+ T-cells capable of inhibiting HIV-1 replication and identified epitopes targeted by broadly potent and effective HIV-1-specific CD8+ T-cells. This approach will now be used to evaluate antiviral T-cell responses elicited by candidate HIV-1 vaccines.

Taken together, these reports emphasize the significant role of cell-mediated immunity in host-virus interactions and highlight the need for additional efforts to incorporate CD8+ T-cells into strategies for HIV-1 prevention and functional cure.

## Author contributions

BS wrote the first manuscript draft, JD and MB edited it, and all authors revised it. All authors listed have made a substantial, direct, and intellectual contribution to the work, and approved it for publication.

